# Editorial: Viral impact on CNS: mechanisms of immune dysfunction and cognitive decline

**DOI:** 10.3389/fimmu.2025.1639948

**Published:** 2025-06-17

**Authors:** Thomas A. Angelovich, Sonia Mediouni, Robyn S. Klein, Jacob D. Estes, Bruce J. Brew, Melissa J. Churchill

**Affiliations:** ^1^ ATRACT Research Centre, School of Health and Biomedical Sciences, Royal Melbourne Institute of Technology (RMIT) University, Melbourne, VIC, Australia; ^2^ Department of Infectious Diseases, The University of Melbourne at the Peter Doherty Institute for Infection and Immunity, Melbourne, VIC, Australia; ^3^ Life Sciences, Burnet Institute, Melbourne, VIC, Australia; ^4^ Department of Immunology and Microbiology, The Herbert Wertheim UF Scripps Institute for Biomedical Innovation & Technology, Jupiter, FL, United States; ^5^ Department of Microbiology & Immunology, University of Western Ontario, London, ON, Canada; ^6^ Vaccine & Gene Therapy Institute, Oregon Health & Science University, Beaverton, OR, United States; ^7^ Peter Duncan Neurosciences Unit, Departments of Neurology and Immunology St Vincent’s Hospital, Darlinghurst, NSW, Australia; ^8^ Faculty of Medicine, University of New South Wales, Sydney, NSW, Australia; ^9^ Departments of Microbiology and Medicine, Monash University, Melbourne, VIC, Australia

**Keywords:** brain, viruses, neuroinflammation, neuropathology, cognition

## Introduction

Recently, the contribution of viruses to neuropathology and cognitive decline has garnered significant interest with viral infection associated, at least in part, with the pathogenesis of dementia, multiple sclerosis and virus-specific cognitive impairment (1–4). Neuropathology can occur during acute, chronic and latent infection and, in some cases, even in the presence of antiviral therapy. However, the precise mechanisms by which specific viruses induce neuropathology and cognitive dysfunction remain unclear. This underscores the need to elucidate the underlying processes in order to develop effective therapeutic strategies. In this Research Topic, we have collated a series of manuscripts that assess the contribution of various viruses including Human Immunodeficiency Virus (HIV), Severe acute respiratory syndrome coronavirus 2 (SARS‑CoV‑2), and others, to neuroinflammation, neuropathology and cognitive disorders.

## Summary of contributions

As mentioned above, the role of viruses in neuroinflammation and neuropathology has gained significant attention in recent years. Contributing to this Research Topic, Li and Wu performed a bibliographic analysis of research publications in the field and found an increasing trend in the number of publications and citations over the last 20 years. Naturally, the contribution of pathogens to neuropathology and/or cognitive disorders varies based on factors such as the viral replication cycle, pathogenesis, host responses, and environmental influences. In this Research Topic, Nisa Awan et al. reviewed current evidence regarding viruses contributing to neuropathology and cognitive disorders. They further described underlying processes and, importantly, current clinical trials and drug treatment studies aimed at limiting the impact of viruses on the brain.

Antiviral signaling and cellular activation in the brain are critical defense mechanisms against viral infection, both within the central nervous system and systemically. Several studies in this Research Topic evaluated cellular activation in the context of HIV, revealing notable changes in both *ex vivo* brain tissue and cerebrospinal fluid (CSF) from people with HIV (PWH) - a population that continues to experience neuropathology and, in some cases, cognitive disorders despite viral suppression with antiretroviral therapy (5). Chan et al. demonstrated that the frequency of activated CD4+ and CD8+ T cells increased over time in CSF early during primary HIV infection. While the level of CSF CD4+ T cell activation correlated with levels of CSF HIV RNA, levels of CSF CD8+ T cell activation or the frequency of monocyte subsets in CSF did not. Importantly, T cell activation remained elevated in the CSF following ART initiation, indicating a persistent state of cell activation in the CSF. In a separate study, Byrnes et al. (including members of the editorial team) also demonstrated persistent cell activation in frontal cortex tissue from ART-suppressed PWH. Interestingly, levels of microglial activation were associated with both intact and 5’ defective HIV proviral DNA, supporting a relationship between HIV reservoirs in the brain and neuroinflammation. These findings suggest that ongoing immune activation, driven by viral reservoirs, may contribute to continued neuropathology in PWH despite successful systemic viral suppression.

While HIV is known to directly infect microglia and, to a lesser extent, astrocytes and pericytes, infection can impact surrounding cells including neurons, thereby contributing to neuropathology. Alternatively, signals from surrounding cells may exert protective effects that mitigate neuropathology. In a mini-review, Lopez and Brown described the role and impact of secreted phosphoprotein-1 (SPP1) on innate immune activation and inflammation in the brains of PWH through mammalian target of rapamycin (mTORC1/2) signaling and NLRP3 inflammasome activation to respond to neuronal injury, highlighting a potentially protective crosstalk mechanism between microglia and neurons. Therefore, therapeutic strategies that target sources of persistent inflammation, in combination with earlier treatment initiation, are likely to offer significant benefits in reducing HIV-associated neuropathology.

Importantly, factors beyond viral persistence and/or replication in the brain must also be considered when studying the neuroinflammation and neuropathology associated with viral infections. In a model of systemic viral infections, Li et al. demonstrated that intraperitoneal injection of polyriboinosinic: polyribocytidylic acid (poly I:C), a synthetic analog of viral double-stranded RNA, triggered neuroinflammation in rats. This was measured using [18F]DPA-714 positron emission tomography, supporting the role of peripheral immune activation on central neuroinflammatory processes. Additionally, illicit drug use and other modifiable factors may also exacerbate virus-mediated neuroinflammation. Miao et al. reviewed how methamphetamine use contributes to neuronal activation and persistent HIV in virally suppressed PWH.

A major limitation in studying neuroinflammation and neuropathology is the restricted accessibility of the brain, making direct cellular-level assessment extremely challenging. As a result, identifying predictive biomarkers of cognitive disease and pathology is essential. SARS-CoV-2 infection has been shown to induce long-term neurological effects in some individuals with levels of IL-1β in the brain associated with neuropathology and cognitive impairment (6, 7). In a study of individuals with Long COVID or recovered SARS-CoV-2 infection, Elahi et al. identified a positive correlation between levels of Galectin-9 and artemin with measures of cognitive deficit in people with Long-COVID and myalgic encephalomyelitis/chronic fatigue syndrome, suggesting potential clinical utility as prognostic biomarkers.

Models of viral infection of the brain are also essential tools in understanding the fundamental mechanisms of disease. Current approaches include cell coculture systems, organoids, animal models, and, in some cases, *ex vivo* human organotypic culture; each with distinct advantages and limitations. In a study by Govaerts et al., a mature human pluripotent stem cell (hiPSC)-derived neurospheroid model was used to investigate Varicella-zoster virus infection and antiviral evasion mechanisms. This study demonstrated that these neurospheroids could be infected with VSV and that infection suppressed antiviral signaling, highlighting the model’s potential as a novel platform for studying Vesicular Stomatitis Virus-mediated immune responses.

## Concluding remarks

In summary, this Research Topic offers new insights into the role of viruses as key drivers of neuroinflammation, neuropathology and cognitive impairment, helping to inform future therapeutic strategies.
